# Neonatal Near Miss: the need for a standard definition and appropriate criteria and the rationale for a prospective surveillance system

**DOI:** 10.6061/clinics/2015(12)10

**Published:** 2015-12

**Authors:** Juliana P Santos, José G Cecatti, Suzanne J Serruya, Paulo V Almeida, Pablo Duran, Bremen de Mucio, Cynthia Pileggi-Castro,

**Affiliations:** I Universidade de Campinas (UNICAMP), Faculdade de Ciências Médicas, Departamento de Ginecologia e Obstetrícia, Campinas/SP, Brazil.; II Latin American Center of Perinatology (CLAP), Pan American Health Organization (PAHO), Montevideo, Uruguay.; III Ministério da Saúde, Brasília/DF, Brazil.; IV Universidade de São Paulo, Faculdade de Medicina de Ribeirão Preto, Departamento de Pediatria, Ribeirão Preto/SP, Brazil.

**Keywords:** Neonatal Morbidity, Neonatal Mortality, Neonatal Near Miss, Surveillance, Neonatal Care

## Abstract

In Latin American, there is currently a regional action with the main purposes of putting the concept of severe neonatal morbidity in practice and formulating proposals for interventions. A general overview of neonatal health conditions, including morbidity and mortality, is provided to update regional knowledge on the topic. An example of the development and implementation of the concept of maternal near miss is also provided, followed by results from a systematic review covering all previously published studies on Neonatal Near Miss. Finally, some proposals for building a common concept on the topic and for launching a prospective surveillance study are presented. A Neonatal Near Miss is a neonate who had a severe morbidity (organ dysfunction or failure) but who survived this condition within the first 27 days of life. The pragmatic criteria recommended to be used are as follows: birth weight below 1700 g, Apgar score below 7 at 5 minutes of life and gestational age below 33 weeks. As a proxy for organ dysfunction, the following management criteria are also confirmed: parenteral therapeutic antibiotics; nasal continuous positive airway pressure; any intubation during the first 27 days of life; phototherapy within the first 24 h of life; cardiopulmonary resuscitation; the use of vasoactive drugs, anticonvulsants, surfactants, blood products and steroids for refractory hypoglycemia and any surgical procedure. Although this study starts from a regional perspective, this topic is clearly globally relevant. All nations, especially low and middle-income countries, could benefit from the proposed standardization.

## INTRODUCTION

The Latin American Center of Perinatology (CLAP, Montevideo, Uruguay) from the Pan American Health Organization (PAHO, Washington, DC), with the support from several countries from the region, is currently leading an action with the objectives of filling gaps in knowledge about severe neonatal morbidity and joining expertise on the topic to formulate proposals for interventions, considering that neonatal mortality is currently responsible for approximately 60% of infant mortality in the region. These objectives could contribute to the effort to achieve Millennium Development Goals 4 and 5 until 2015, namely to reduce infant mortality and improve maternal health, respectively 1.

For this purpose, a general overview on neonatal health conditions, including both morbidity and mortality, is necessary to update regional knowledge of the topic. This update should be followed by a round of discussions and proposals with the final aim of building a consensual concept of Neonatal Near Miss. In addition, an environment allowing the performance of a prospective surveillance pilot study in which all Latin American countries can participate is to be created. Although the study starts from a regional perspective, this topic is clearly globally relevant. All nations, especially low- and middle-income countries, could benefit from this standardization.

### Neonatal Near Miss as a tool for reducing neonatal morbidity and mortality in Latin America and the “Stork Network” in Brazil

From 1990 to 2012, infant mortality decreased by 77% in Brazil 2,4. In 2013, the country achieved goal 4 of the Millennium Development Goals by improving infant health conditions in association with public policies and health care programs such as the Unified Health Care System (Sistema Único de Saúde - SUS), thereby augmenting primary health care, the Family Health Program, immunization policies, national maternal breastfeeding policy, Child-Friendly Hospital, Breastfeed Brazil, Milk Bank network and Family Fund Program. This decrease in Brazilian infant mortality was most significant in the poorest regions of the country. Brazil has focused on equity, with the premise that more should be offered to those in greater need, i.e., the north and northeast of the country. Infant mortality rates in these regions are currently close to those in the southeast region, which is the richest in the country 2.

Neonatal mortality in Brazil corresponds to 70% of the total rate of infant mortality; neonatal mortality has reduced more slowly than that of other age groups and has become a major health challenge for the country. The main causes of neonatal mortality in Brazil are prematurity, congenital malformations, perinatal infections and perinatal asphyxia 3. The country has a neonatal resuscitation program supported by the Ministry of Health and coordinated by the Brazilian Society of Pediatrics. A government regulation was designed to ensure that a health professional that is qualified in neonatal resuscitation is in every place of childbirth.

Currently, the perinatal paradox in Brazil is that the causes of high maternal and neonatal mortality rates are preventable with health care actions. In contrast, childbirth has been intensely medicalized, with 98% due to hospitals and 88% due to doctors 2,3. Millennium Development Goal 5 will likely not be achieved by 2015; despite a decrease in maternal mortality, the achievement of this goal remains a challenge. Decreasing the current caesarean rate in the country, which is 52%, also represents a major challenge. Despite a significant reduction in infant mortality, it remains elevated in specific populations such as Indians, *quilombolas* (escaped slaves), riverside populations and others.

The Brazilian Project, named the Stork Network, is aimed at reducing infant and maternal mortality 5,6. Although major technological advances have been made in Brazil, no evidence-based practices have been fully adopted in Obstetrics and Neonatology; thus, unnecessary childbirth and neonatal interventions and the vulgarization of caesarean sections occur. The aims of the Stork Network are to ensure a woman’s right to pregnancy, prenatal care, humane and safe childbirth and postpartum care as well as the newborn infant’s right to safe and humane birth and care. In addition, investment has been made in infant development such as in the Tender Brazil Program and programs existing in Chile, Colombia and Cuba. The Stork Network acts in the prenatal, childbirth and postpartum phases, in family planning and in infant care until 2 years of age via making information available, mobilizing society, creating committees and investing in collective spaces. Other aims of the network are greater autonomy for the woman in labor, vulnerability and risk assessment, risk classification, educational practices and action by the obstetric nurse in a multiprofessional team. The network ensures that laboratory tests are conducted at the appropriate time, links basic care to maternity hospitals and informs the mothers of the place of childbirth. The network aims to increase the number of neonatal beds, mainly in those regions in greater need, investing in the Kangaroo Method to capacitate professionals. Furthermore, promoting the presence of the father from prenatal care to birth and postpartum period follow-up and creating childbirth centers with obstetric nurses to perform deliveries and pregnant mother-baby house are other aims.

From 2009 to 2011, Brazil made advances in a surveillance method for maternal and infant deaths with a rigid protocol for notification, investigation and recording. This method may help in the implementation of any action involving Neonatal Near Miss.

### Neonatal Health in Latin America and the Caribbean: problems and challenges

In the region of the Americas, there are 15.6 million births annually. Of these births, 94% are assisted by skilled health professionals. Of the total number of births, 200 thousand infants die before the first year of life, and 125 thousand infants die in the first 28 days of life, accounting for neonatal deaths 3,4.

Between 1990 and 2010, neonatal mortality decreased by approximately 50%. However, neonatal mortality was the component that decreased the least among children under 5 years of age. Neonatal mortality contributed 43% in 1990 and 50% in 2010 to the mortality rate in those under 5 years of age 7,8.

In an analysis of neonatal mortality, from 60 to 70% of infant deaths occurred before 7 days of life. Additionally, participation of the neonatal component in mortality varied widely in those under 5 years of age in different countries in the Americas. Mortality in those under 5 ranged from < 10 to 100 deaths per thousand live-born infants among American countries. This great variation also occurred in different regions within each country. Prematurity (35%), neonatal asphyxia (15%) and infections (12%) represent the main causes of death in Latin America and the Caribbean, accounting for 3/4 of all the causes of neonatal mortality. These percentages are similar in all the regions of the Americas and are important markers of neonatal mortality and morbidity not only after birth but also in prenatal and perinatal care 4,9.

Annually, approximately 1.2 million newborn infants weigh <2500 g at birth and approximately 1.2 million are premature. The majority of newborn infants, almost 1 million, are born between 33-36 weeks; 80 thousand are premature infants born under 28 weeks. The extremely premature infants have the greatest risks and are fewer in number. However, the remaining premature infants are also at risk.

Approximately 1 million newborn infants in the Americas require some maneuver at birth. Resuscitation maneuvers are dependent on the quality level of the health facility. Procedures involved in resuscitation range from aspiration, oxygenation and intubation up to admission to the neonatal intensive care unit (ICU). The mortality risk is 5- to 20-fold higher in premature infants, with short-term risks of neurological alterations, thermal dysregulation and metabolic dysregulation. Some may present visual and hearing alterations, chronic pulmonary disease, and neurological deficits with medium to long-term consequences in quality of life; there may also be a family and a social impact. Retinopathy is a consequence of prematurity, the quality of perinatal care and the use of oxygen. The prevalence of retinopathy in newborn infants in the region ranges from 20 to 30% in those with a birth weight <1500 g, meaning that 1 in every 3 very low birth weight infants has a risk of retinopathy. This aspect is important to address in the quality of care 10, as the prevalence of blindness due to prematurity retinopathy ranges from 13% to 60% in the region 11.

Late preterm infants represent an average number of 1 million infants born in the region. There is a 1.2- to 3-fold higher risk of some morbidity, respiratory difficulty, jaundice and the need for endovenous fluids, antibiotics and certain interventions in infants born at 37-38 weeks compared with infants born at 39 weeks. From a health management perspective, this reflects on increased health care costs. The cost of preterm infant care is estimated to be approximately US$ 10 million per year.

In 2008, the PAHO/CLAP developed a strategy and regional action plan for newborn health in the context of a continuous process of maternal, newborn and infant care, considering that the neonatal component had insufficient visibility in the political agenda. Specific action plans to address the neonatal topic and disaggregated data remained limitations. It was only possible to work with national mean values. Furthermore, the quality of care was impaired in the implementation of evidence-based interventions and limited access to health care was available 12.

The action plan was created with the purpose of providing countries of the region with support to achieve the Millennium Development Goals, highlighting the interventions to be implemented in perinatal health care, guided by 4 strategic areas: 1) create a favorable environment for the promotion of neonatal health; 2) strengthen health care systems and improve access to health care services for women, newborn infants and children; 3) promote community interventions; 4) create and strengthen systems for the surveillance, monitoring and classification of specific topics linked to the newborn infant 12.

After 5 years since the approval of this regional action plan, a medium-term analysis showed that 9 in every 10 countries have a national plan for maternal and neonatal health care. However, only 57.1% have a specific budget for neonatal care and 82% of the countries have strategic alliances; such alliances were considered important or very important. Regarding access to quality health care, 99% of births are assisted by skilled professionals in more than half of the countries and this number is lower than 90% in 20% of the countries. However, in some geographical areas, coverage is lower than 50% and even lower than 10%. The norms, guidelines and protocols are already available, but their implementation is often limited and may vary from 40% to 100% 9.

Countries also have information systems with varied and often limited coverage and reach; 88.5% have health systems that evaluate vital signs, 77% have health information systems (SIP/CLAP) and 70% have surveillance systems of mortality. Health information systems are less common at the community level (50%).

Based on these limitations and difficulties, focus should be directed toward development of the neonatal health component, as follows: 1) short-term: maintain efforts to reduce neonatal mortality and intervene in the main causes of preventable death, especially prematurity; 2) medium-term: address topics that contribute to a reduction in morbimortality and that affect quality of life, such as prematurity, metabolic conditions, development and growth, to improve quality of life; 3) and long-term: strengthen community experiences or interventions that produce optimal results. These strategies are developed with technical cooperation from countries and through the generation of evidence, the formulation of guidelines and capacitation.

The strategy approach is to focus on the priority aspects associated with morbidity and mortality, to strengthen health services, to work on the capacitation of human resources and to articulate with other groups contributing to experience for technical development. With the knowledge of neonatal mortality, new challenges may be discussed, seeking a surveillance system of severe neonatal conditions that require decision-making in the search for better results in neonatal care.

### Development of the concept of Maternal Near Miss as an example

Understanding of the development process of the concept and criteria of Maternal Near Miss may help in the development of Neonatal Near Miss. In 2009, the definition of Maternal Near Miss (MNM) was published. Women suffering a severe complication during pregnancy, childbirth or within 42 days of the postpartum period, at risk of death, but survived by chance or due to effective interventions were considered maternal near miss 13.

The original idea, in the context of a public policy, was to strengthen health quality through a surveillance system by which severe maternal morbidity could be identified through hospital and mortality information systems. The aim was to create an online system for identifying severe morbidity cases, raising an alert and treating the case as priority, initiating a series of interventions at the location or transferring the patient to a place where adequate care could be given.

A systematic review was the first step in the development of Maternal Near Miss in Brazil and worldwide 14,15. Few studies had previously been published on the concept. In the second half of the decade of 2000, the study sought optimal criteria for severe maternal morbidity based on the Mantel criteria (South Africa), which were fundamental dysfunctions of organs and systems and the Waterstone criteria (England) using clinical criteria and combined criteria 16,17. None of the criteria alone were sufficiently specific to define a population at risk.

Questionnaires that were validated and used in the research on the World Health Organization (WHO) Global Survey were applied to construct a pragmatic definition of Maternal Near Miss. The pragmatic criteria used were retrospective because they were easily collected. The management criteria and organ dysfunctions were based on case management. The most common criteria in Maternal Near Miss cases were hysterectomy, admission to the ICU, blood transfusion, cardiac or renal complication and eclampsia. In subsequent studies, cardiac or renal complications were not confirmed. The pragmatic criteria established by the WHO are currently hysterectomy, admission to the ICU, blood transfusion and eclampsia 18.

Due to the diversity of criteria for Maternal Near Miss, women were interviewed by population surveys to provide information about the occurrence of any severe complications. The criteria for severe maternal morbidity were tested in a questionnaire answered by women through a module introduced in the Brazilian National Demography Health Survey (PNDS) on maternal morbidity. The pragmatic criteria defined by the WHO were tested, finding 21 cases /1,000 live births (LB), the first estimate of the occurrence of Maternal Near Miss in the Brazilian population. The results were published, confirming the criteria for eclampsia, hysterectomy, admission to the ICU and blood transfusion, which together determined effectively the total number of maternal deaths 19.

Severity scores were tested and those addressing dysfunction or failure showed optimal results. Criteria concerning the organ systems were used. These criteria were elaborated in such a manner that they could be identified in different settings, both in tertiary services that have laboratory markers available and in primary services, along with corresponding clinical dysfunctions. The idea was to produce a system that could be used prospectively, including a group of clinical situations that could potentially define a threat to life. A checklist would be used for this purpose, to observe a group of other variables that would also indicate a threat to life in a more specific manner. Therefore, Near Miss is always a retrospective diagnosis and cases of death are similar; the only difference is the actual outcome, which occurs by chance or by an effective intervention.

In developing the WHO criteria, the Sequential Organ Failure Assessment (SOFA) was tested between 2002 and 2006 in all women with potentially life-threatening conditions from a Brazilian maternity. The study showed that SOFA performed better among organ failure and death. This pre-validation of the criteria for organ dysfunction showed highly satisfactory results 20. Therefore, the WHO also included these dysfunction criteria. In 2009, there was a publication on the definition and criteria of Maternal Near Miss established by the WHO 13.

After the criteria were tested, the WHO recommended that the approach to identify Maternal Near Miss, with several indicators constructed from surveillance, should qualify the type of health care provided. In Brazil, a national surveillance network for severe maternal morbidity was constructed, including 27 centers. During one year, a prospective surveillance of all criteria for potentially life-threatening conditions was conducted 21. This surveillance generated a maternal severity index for transforming collected data into scores, allowing the quality of obstetric care to be assessed. This index was used in the study of the WHO Multicountry Survey that included over 300 thousand cases to identify the quality of obstetric care in three continents 22. A delay in seeking and reaching the facility was frequently observed, as was a delay in receiving the adequate type of care. Delays were directly associated with outcome severity and occurred in 52% of potentially life-threatening conditions, in 68% of Maternal Near Miss cases and in 84% of maternal deaths, indicating the accountability of hospital managers and health care professionals 23.

### Systematic Review on Neonatal Near Miss

Analogous to Maternal Near Miss, the proposed definition of Neonatal Near Miss would be a newborn infant who nearly died but who survived a complication occurring during pregnancy, during childbirth, or in the first 7 days after the termination of pregnancy. The criteria to be used should be simple, highly correlated with death, sufficiently rare and homogeneous for mortality risk in each case. A Neonatal Near Miss case should be strikingly similar to a death case because the two are similar, except for the outcome. The Neonatal Near Miss criteria were also based on the identification of markers of organ dysfunction, including laboratory, management and clinical criteria, and on interventions associated with the management of severity.

A systematic review was performed following the Preferred Reporting Items for Systematic Reviews and Meta-Analyses (PRISMA) guidelines for systematic reviews 24. Three electronic databases were consulted: EMBASE, PubMed and SciELO. The keyword “Neonatal Near Miss” was used. The search syntax was as follows: “(neonatal morbidity) AND (neonatal illness severity score) OR (neonatal diseases severity score) AND (neonatal mortality)”. No restrictions on time or language were used and all types of studies were accepted. One hundred and eight (108) articles were initially found in these electronic databases. Duplicate articles, irrelevant titles and full-text articles containing no original Neonatal Near Miss data were excluded. Four articles were selected for review 25.

The first study, entitled “Neonatal Near Miss: a measure of the quality of obstetric care”, was published in 2009. In this study, data from “Saving Babies: 2003-2005: Fifth Perinatal Care Survey of South Africa” were used 26. For criteria to define Neonatal Near Miss, markers of dysfunction and/or organ failure were used, showing an infant mortality rate of 6.3/1,000 LB and a Neonatal Near Miss rate of 24.7/1,000 LB. The majority of Neonatal Near Miss cases were of dysfunction/respiratory failure (63%), followed by dysfunction/immunologic failure, including infections (21.2%), and by dysfunction/central nervous system failure (5%). Compared with neonatal deaths, more cases of Neonatal Near Miss were observed in obstetric patients in whom asphyxia, trauma or antepartum hemorrhage had occurred. According to these data, many morbidity cases are lost when only neonatal mortality cases are evaluated.

The second study, “Neonatal Near Miss approach in the 2005 WHO Global Survey Brazil”, was published in 2010. After cleaning the database, 15,169 cases were used. In this study, a secondary analysis with the Brazilian component of the “2005 WHO Global Survey on Maternal and Perinatal Health” database was performed 27. A definition of Neonatal Near Miss was developed based on the main causes of death: prematurity and perinatal asphyxia. The conditions associated with a risk of Neonatal Near Miss were very low birth weight (<1500 g), a gestational age younger than 30 weeks at birth and an Apgar score lower than 7 at 5 minutes of life. The early neonatal mortality rate was 8.2/1,000 LB and the Neonatal Near Miss rate was 21.4/1,000 LB. The rate of early neonatal mortality was estimated by the percentage of deaths in the first week of life among newborns at risk during delivery (27.7%). The severe neonatal outcome rate, which was 29.5/1,000 LB, was calculated by the sum of the number of deaths and the number of Near Miss cases. In this study, 121 death cases were observed; 80% of these cases had some of the selected conditions. Diagnostic evaluation was the adopted approach: the Near Miss criteria were considered diagnostic tests for mortality prediction. With these criteria (gestational age <30 weeks, very low birth weight or Apgar <7 at the fifth minute of life), the sensitivity was 82%, the specificity was 97%, and almost all of the cases had information available in medical charts. Comparing the different services revealed that Neonatal Near Miss could also be used to evaluate the quality of perinatal care.

The third study was published in 2014 with the title “Development of criteria for identifying neonatal near-miss cases: analyses of two WHO multicountry cross-sectional studies”. Secondary analyses of the following two WHO databases were performed: the “Global Survey on Maternal and Perinatal Health” (WHOGS) and the “Multicountry Survey on Maternal and Newborn Health” (WHOMCS). In the first database, 277,706 cases were analyzed, revealing a mortality rate of 1.35%. In the second study, 309,644 cases were analyzed, revealing a mortality rate of 1.08%. In the Global Survey database, criteria showing homogeneity in terms of the mortality risk were chosen and the optimum combination of diagnostic accuracy was obtained with an Apgar score <7 at 5 minutes, a gestational age <33 weeks and a birth weight <1750 g. These criteria were validated and had the same performance when applied to the Multicountry Survey database. The total early neonatal mortality rate was 7.4/1,000 LB and the Neonatal Near Miss rate was 44.4/1,000 LB 28.

Adopting a different approach than WHOGS, the WHOMCS used specific data on the management of neonatal severe morbidity in addition to the three previously mentioned variables. The following markers of severity management were based on interventions used in the South African study: the use of intravenous antibiotics, the use of nasal continuous positive airway pressure (CPAP) or intubation at any time in the first week of life, cardiopulmonary resuscitation, the use of any vasoactive drug, the use of phototherapy in the first 24 hours of life, the use of anticonvulsants, the administration of a surfactant, the use of blood transfusion, the use of corticoids in refractory hypoglycemia, and any surgery in the first week of life 29. The only stratifying variable used was the human development index (HDI) from 2012.

In this study, countries with a moderate and low HDI predominated. The pragmatic criteria alone showed a sensitivity of 77%, a specificity of 96% and a diagnostic odds ratio of 87. The management criteria showed a sensitivity of 79%, a specificity of 94% and a diagnostic odds ratio of 66. The combination of pragmatic criteria and management criteria showed a better performance with a sensitivity and specificity of almost 93% and a very good diagnostic odds ratio of 163.

The total rate of early neonatal mortality was 9.2/1,000 LB and the Neonatal Near Miss rate was 72.5/1,000 LB considering any pragmatic or management marker. The Neonatal Near Miss rate was 37.4/1,000 LB for pragmatic markers and 53/1,000 LB for management markers. The neonatal mortality index was 12.7% and the ratio of severe neonatal outcome rate was 81.7/1,000 LB. Based on these results, the optimal criteria for severe morbidity prediction were intubation in the first week of life, cardiopulmonary resuscitation and the use of vasoactive drugs. The case-fatality relationship and the mortality index were also evaluated, showing that countries with a high HDI had a high case-fatality relationship and a low mortality index; the opposite was observed for countries with a low HDI 28.

The fourth study, titled “Neonatal near miss in the Birth in Brazil Survey”, was published in 2014 using data from the Born in Brazil Network. Nineteen variables were used: Apgar <7 at 5 minutes of life, gestational age (≤32, 33 to 36 and ≥37 weeks), birth weight (<1500, 1500 to 2499 and ≥2500 grams), twinning, the use of mechanical ventilation, the use of oxygen supplementation after birth, admission in the neonatal intensive care unit (NICU), the use of nasal CPAP, intubation in the delivery room, cardiac massage, the use of resuscitation drugs, phototherapy in the first 72 hours of life, the use of a surfactant, the use of antibiotics in the first 48 hours of life, congenital malformation, seizures, newborn respiratory disease (transitory tachypnea, hyaline membrane disease, pulmonary hypertension or meconium aspiration syndrome), hypoglycemia and necrotizing enterocolitis. The neonatal mortality rate was 11.1/1,000 LB and the Neonatal Near Miss rate was 39.2/1,000 LB. Variables associated with neonatal death were birth weight <1500 g, Apgar <7 at 5 minutes, the use of mechanical ventilation, premature infant <32 gestational weeks and newborn infants with congenital malformation 30.

Despite similarities between the criteria such as prematurity and asphyxia chosen for each study, the criteria were not the same because there remain no internationally accepted definition and criteria. The neonatal period used in each study ranged from 3 to 28 days after birth. All studies analyzed showed that the Neonatal Near Miss rate was higher than the neonatal mortality rate, increasing 2.6- to eight-fold.

A systematic review is the first step in validating the concept and identifying useful indicators to explore quality of care, establishing priorities in the management of these newborn infants, improving neonatal health care and thus reducing the negative impact on the future lives of these children. The criteria for defining the concept should be simple, applicable in individual services and at the health system level and significant for doctors, hospital administrators and health care professionals.

### Criteria for identifying Neonatal Near Miss cases developed from WHO studies

The proposal regarding Neonatal Near Miss is to seek an instrument to evaluate the quality of perinatal care. The proposal concerns not only with the neonatal period but with also obstetric care and seeks to identify opportunities for health improvement at the population level and possibilities to assess more informative indicators than neonatal mortality.

The development of Neonatal Near Miss concept and criteria may allow comparisons of the same institution over time and of different institutions from different regions. Discussions of morbidity are better accepted than death case reviews by health care teams. Thus, the quality of care would improve. There is an expectation of the development of prospective surveillance processes of severe neonatal morbidity. The problem is that until now, a large number of facilities, especially tertiary level services use complex risk assessments that require laboratory support such as SNAPPE and CRIB 31,32.

The Global Survey study was performed between 2004 and 2008, collecting hospital information on care provided upon a woman’s admission until the newborn infant was 7 days old. Institutional clustering and multistage sampling methods were used, with a probability proportional to the population size. Each institution should perform at least 1,000 deliveries annually and have the capacity to perform caesarean sections. These criteria are only met in more complex services 18,28. Preliminary results of an ongoing analysis of the WHO Multicountry Survey database guided the selection of the proposed criteria for case definition.

There is concern regarding development of the concept of Neonatal Near Miss and which direction that it should follow. It could be restricted to neonatal care in the ICU, but several previously validated and accepted scores are known to be very useful for this purpose. Peripartum and intrapartum care as well as the early neonatal repercussions could also be assessed. Considering that the construction of Neonatal Near Miss is to prevent death by effective interventions, the role of extreme preterm and congenital malformations should be more seriously discussed in this context.

### A step forward: proposal for a common definition, criteria, related health indicators and rationale for a prospective surveillance system

The development of criteria and the definition of Neonatal Near Miss are important for the subsequent creation of an epidemiological surveillance system to be used as a tool for public policy and case management. For this purpose, criteria must be initially held on databases. However, a comprehensive and prospective pilot study should be subsequently conducted. The study may start with a sentinel surveillance system in locations with a large number of births using preexisting databases. A validation study should be conducted to test whether the criteria may be generalized.

The data form the construction process and the application of a pilot study to the Maternal Near Miss model performed by CLAP is important to guide a similar procedure for the Neonatal Near Miss condition. The Maternal Near Miss form includes maternal data, clinical and laboratory criteria and interventions. Data were collected by trained individuals and subsequently crosschecked. Some health services could identify the problem through the form. However, these services had no idea of what to do with the information. The concept is that other locations may monitor the occurrence of an event by using a computerized system. Based on evidence of a problem, searching for strategies and then performing the adequate evidence-based intervention recommended for each situation is possible.

The definition of Neonatal Near Miss is “a newborn infant, classified by severe morbidity (Near Miss) assessment criteria, who survived these conditions within the first 27 days of life”. For the identification of Neonatal Near Miss, two groups of criteria were established based on the results of previous studies on the topic. The first was formed by the following pragmatic criteria defined:

- Birthweight < 1750 g
- Apgar score <7 at 5 minutes
- Gestational age < 33 complete weeks

The second group was characterized by the following management criteria:

- Parenteral antibiotic therapy (up to 7 days and before 28 days of life)
- Nasal CPAP
- Any intubation up to 7 days and before 28 days of life
- Phototherapy within 24 hours of life
- Cardiopulmonary resuscitation
- Use of vasoactive drugs
- Use of anticonvulsants
- Use of surfactant
- Use of blood products
- Use of steroids for the treatment of refractory hypoglycemia
- Surgery

Some management variables were not analyzed in previous studies; because these variables may be important to characterize a Neonatal Near Miss case, they should be tested in future studies:

- Use of antenatal steroid (categorize treatment regimens)
- Use of parenteral nutrition
- Identification of congenital malformation (classify types/severity groups according to the International Classification of Diseases, 10th revision – ICD-10)
- Apgar score at 5 minutes, if considered a Near Miss case by another criterion
- Admission to the ICU

Although congenital malformation cases performed well as severity markers in some studies, many of these death cases may not have been preventable even with effective interventions. Therefore, evaluating quality of care would not be possible. The same situation should be questioned and assessed regarding extremely premature infants.

Considering this concept and definition as well as the set of criteria to be used for the identification of Neonatal Near Miss, the corresponding health indicators with their respective definitions are as follows:

1. **Early neonatal mortality rate (ENMR):** refers to the death of a live-born infant within the first 6 days of extrauterine life/1,000 LB.
2. **Neonatal mortality rate (NMR):** refers to the death of a live-born infant within the first 27 days of extrauterine life/1,000 LB.
3. **Neonatal Near Miss rate (NNMR):** (number of Neonatal Near Miss cases/total number of LB) X 1,000.
4. **Early severe neonatal outcome rate (ESNOR):** (number of early neonatal deaths + number of early Neonatal Near Miss cases/total number of LB) X 1,000.
5. **Severe neonatal outcome rate (SNOR):** (number of neonatal deaths + number of Neonatal Near Miss cases/total number of LB) X 1,000.
6. **Case-fatality ratio:** refers to the ratio between the number of Neonatal Near Miss cases per neonatal death case.

A specific chart for data collection should be concomitantly developed with the initial study protocol design. All health facilities providing childbirth care in Latin America and the Caribbean should participate in the study. The selection of participating centers is to be performed by countries in those regions, considering a representative sample of all levels of care. Hopefully this article will trigger a global rising interest in the topic and incur broader participation of countries from all over the world in the study, resulting in this concept and these criteria being used as a true tool for improving the quality of health and life of neonates worldwide.

### PAHO Neonatal Near Miss Working Group

**Argentina:** Alejandro Jenik (Hospital Italiano de Buenos Aires); **Brazil:** Antonio Moura da Silva (Federal University of Maranhão, São Luis), Francisco Eulogio Martinez (Ribeirão Preto Medical School, University of São Paulo), Joao Henrique C. Leme de Almeida (Instituto Fernandes Figueira, Fiocruz, Rio de Janeiro), José Simon Camelo Jr (Ribeirão Preto Medical School, University of São Paulo), Oscar Suriel (Pan American Health Organization, Brazil), Roseli Calil (University of Campinas), Sergio Tadeu Martins Marba (University of Campinas), Tatiana Raquel Selbmann Coimbra (Brazilian Ministry of Health, Brasilia), Zeni Carvalho Lamy (Federal University of Maranhão, São Luis); **Colombia:** Jorge Mejia Lopez (Del Valle University, Cali); **Peru:** Marisol Vicuña Olivera (Colectivo Neonatal, Lima); **USA:** Arnoldo Grosman (University Maimonides, Washington), Goldy Mazia (Latin American and Caribbean Neonatal Alliance); **Uruguay:** Gerardo Martinez (CLAP, PAHO, Montevideo), Magdalena Bonasso (CLAP, PAHO, Montevideo), Rodolfo Gomez Ponce de Leon (CLAP, PAHO, Montevideo).

## AUTHOR CONTRIBUTIONS

The idea for this manuscript arose from a discussion among Serruya SJ, Duran P and Mucio B. Santos JP and Cecatti JG wrote the first version of the manuscript, with input from Almeida PV, Duran P, Mucio B and Pileggi-Castro C. All authors read and agreeded on the content of the current version.

## ACKNOWLEDGMENTS

The current plan of action was sponsored by the CLAP/PAHO and the Brazilian Ministry of Health. It was originally discussed in a regional meeting that was held in São Paulo, Brazil, in May 2014.
